# Breeding Diploid F_1_ Hybrid Potatoes for Propagation from Botanical Seed (TPS): Comparisons with Theory and Other Crops

**DOI:** 10.3390/plants11091121

**Published:** 2022-04-21

**Authors:** John E. Bradshaw

**Affiliations:** Honorary Associate, James Hutton Institute, Dundee DD2 5DA, UK; johnbradshaw949@btinternet.com

**Keywords:** inbreeding depression, introgression, hybrid vigor, planting material, quantitative traits, recessive mutations, self-compatibility

## Abstract

This paper reviews the progress and the way ahead in diploid F_1_ hybrid potato breeding by comparisons with expectations from the theory of inbreeding and crossbreeding, and experiences from other diploid outbreeding crops. Diploid potatoes can be converted from an outbreeding species, in which self-pollination is prevented by a gametophytic self-incompatibility system, into one where self-pollination is possible, either through a dominant self-incompatibility inhibitor gene (*Sli*) or knockout mutations in the incompatibility locus. As a result, diploid F_1_ hybrid breeding can be used to produce genetically uniform potato cultivars for propagation from true potato seeds by crossing two near-homozygous inbred lines, derived from a number of generations of self-pollination despite inbreeding depression. Molecular markers can be used to detect and remove deleterious recessive mutations of large effect, including those in tight repulsion linkage. Improvements to the inbred lines can be made by introducing and stacking genes and chromosome segments of large desirable effect from wild relatives by backcrossing. Improvements in quantitative traits require a number of cycles of inbreeding and crossbreeding. Seed production can be achieved by hand pollinations. F_1_ hybrid planting material can be delivered to farmers as true seeds or young plants, and mini-tubers derived from true seeds.

## 1. Introduction

The potato (*Solanum tuberosum*) is the world’s fourth most important food crop after maize, rice, and wheat, with 359 million tonnes fresh weight (FW) of tubers produced in 2020 from 16.5 million hectares of land, in 163 countries, resulting in a global average yield of 21.8 t ha^−1^ (http://faostat.fao.org, accessed on 4 January 2022). Most cultivated potatoes are tetraploids, and their ancestry can be traced back to autotetraploids (2*n* = 4*x* = 48) of the diploid cultigen *S. tuberosum* Stenotomum Group (2*n* = 2*x* = 24), which was domesticated from diploid wild species in the highlands of Southern Peru [1], from at least 7000 years before present [2]. Furthermore, most cultivated potatoes are vegetatively propagated and grown from tubers. However, when the International Potato Centre (CIP) was founded in Lima, Peru, in 1971, propagation by true potato seed (TPS) appeared an attractive proposition for the torrid zones of the lowland tropics and subtropics. By 1994, Golmirzaie et al. [3] thought that TPS technology could be the basis of a new green revolution, designed specifically to improve the production and consumption of potatoes in the developing countries of the torrid zone. Later crop maturation and less genetic uniformity could be outweighed by the reduced costs of planting material (TPS), flexibility of planting time, and freedom from tuber-borne diseases [3]. In contrast, when Chilver et al. [4] reviewed on-farm profitability and prospects for TPS, they concluded that widespread geographic adoption was unlikely in the immediate future, but that investment in a small but sustained TPS breeding effort could be justified in both China and India. However, there has been renewed enthusiasm for TPS technology since the advent of diploid F_1_ hybrid breeding in 2008 [5], as it made possible the production of genetically uniform cultivars. More generally, Jansky et al. [6] and Jansky and Spooner [7] have argued that the next step in the development of potato breeding may be a return to the diploid level to implement an inbred-line-based strategy leading to F_1_ hybrid cultivars. This would allow potato breeders to reduce the genetic load of undesirable alleles through inbreeding and then combine desirable traits through hybridization, as also pointed out by Bachem et al. [8].

It therefore seems timely not only to review progress in diploid F_1_ hybrid potato breeding, but also to make comparisons with expectations from the theory of inbreeding and crossbreeding, and with the experiences from other crops, such as the ones I covered in my book on plant breeding [9]. First, however, it is worth reminding ourselves that the scientific foundations for these considerations can be found in the work of Mendel, Darwin, and Fisher.

## 2. Mendel, Darwin, and Fisher

Darwin studied the effects of cross- and self-fertilization in 57 species of flowering plants from 52 genera and 30 families [10]. He conducted experiments over 11 years, involving up to 10 successive generations of self-pollination and the growing of offspring in pots in his greenhouse or in rows in his garden. His most important conclusion was that cross-fertilization is generally beneficial and self-fertilization (inbreeding) injurious, and hence the reason for the existence of mechanisms to promote cross-pollination. He did, however, find that some species were tolerant to inbreeding, although crosses between cultivars displayed hybrid vigor. One such species was the garden pea (*Pisum sativum*), chosen by Mendel for his experiments in plant hybridization that led to his laws of inheritance [11]. Mendel’s work was unknown to Darwin, and remained unknown to the world until rediscovered in 1900, translated into English in 1901, and promoted by Bateson [12]. In retrospect, the work of Mendel on the mechanism of inheritance, and of Darwin on the mating system, can be viewed as the foundations of scientific plant breeding from the beginning of the 20th century. The world’s four most important food crops have three different mating systems. Wheat (*Triticum aestivum*) and rice (*Oryza sativa*) are inbreeding, seed-propagated crops; maize (*Zea mays*) is an outbreeding, seed-propagated crop; and the potato (*S. tuberosum*) is an outbreeding, but primarily clonally propagated, tuber crop. It can be argued that, as a consequence, maize breeding has benefited the most, and potato breeding the least, from underpinning genetics research [13]. Darwin was particularly interested in the evolutionary advantages and disadvantages of the variation found in breeding and mating systems, and how one system could evolve into another, such as the shift from crossbreeding to inbreeding [10,14]. Fisher combined the work of Darwin and Mendel into a theory of inbreeding, with the important addition of an explanation for disadvantageous genes (alleles) tending to be recessive [15]. Furthermore, he advocated greater use of inbreeding for the practical improvement of domestic plants and animals, given the resounding success of the methods adopted for maize improvement based on inbreeding. Indeed, one of the major achievements in 20th century plant breeding was the production of high yielding, genetically uniform F_1_ hybrid cultivars of outbreeding crops from genetically variable, open-pollinated ones. Thus, let us return to the breeding of diploid potatoes, starting with diploid potato germplasm.

## 3. Diploid Germplasm

Andean farmers still cultivate diploid landraces and named cultivars of the Stenotomum Group, the Goniocalyx Subgroup, and the more widely grown Phureja Group, of *S. tuberosum* [16]. Such germplasm is currently proving valuable for the genetic biofortification of potatoes with iron and zinc [17,18,19]. Furthermore, from the 1960s, potato breeders in North America and Europe successfully widened the genetic base of their breeding programs through the selection of long-day-adapted diploid populations of Phureja/Stenotomum Groups from landraces held in genebanks [20,21,22]. Diploid cultivars have been produced from this germplasm such as Mayan Gold, Inca Sun, Inca Dawn, Mayan Queen, Mayan Star, and Mayan Twilight, as part of a program in Scotland [23].

Since the 1960s, it has also been possible to reduce tetraploid *S. tuberosum* to the diploid level through crosses with 2*x* Phureja Group ‘pollinators’ that induce dihaploid production by a form of parthenogenesis [24,25]. Subsequently the frequency of dihaploids (also referred to as haploids) has been increased by the use of elite pollinators, such as IVP35, IVP48, IVP101, and PL-4 [26,27], although the frequency is still low, with reported values of 4.6 to 25.6 dihaploids per 100 berries [28]. Busse et al. [28] have described a high throughput method of production based on the original method of Peloquin and Hougas. However, dihaploids are usually male-sterile, unlike two of the first four, US-W1 and US-W4 [29,30], and female fertility can also be a problem in breeding [28]. Most dihaploids, like the cultivated diploid groups, display gametophytic self-incompatibility [31,32], which prevents the production of true breeding lines by self-pollination. In other words, diploid potatoes are outbreeders, and the same is true for most of their diploid wild relatives. The self-incompatibility (*S*) locus was mapped to chromosome 1 by Gebhardt et al. [33] and Kaufmann et al. [34].

In summary, there is no shortage of diploid germplasm available for diploid F_1_ hybrid breeding, but, as in all breeding programs, it will be important to use the best germplasm available for a particular target environment and particular end use. Breeding objectives will no doubt be expressed in terms of improving yield potential and tolerance to important abiotic stresses, and improving quality traits and resistance to important pests and diseases.

## 4. Breeding Methods for Diploid Potatoes

**Figure 1:**
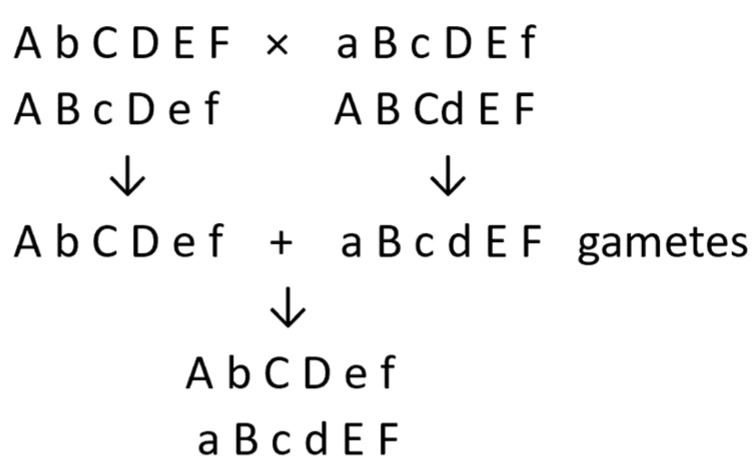
New cultivar from cross between two heterozygous diploid parents (one pair of chromosomes shown, where small letters represent deleterious recessive alleles).

Breeding diploid potatoes for clonal propagation can be conducted through the testing, selection, and vegetative multiplication of desirable clones from population improvement schemes, such as mass or family selection [17,18,19,20,21,35], or from pair crosses between clones that complement each other for desirable traits [23]. The new cultivar will have come from combining a gamete from its female parent with one from its male parent. For each of the 12 pairs of chromosomes, the female-derived chromosome will need to complement the male-derived chromosome for genetic loci at which they have different alleles. In the following simple example, small letters represent deleterious recessive alleles (Figure 1).

Thus, in the selected genotype, as in the parents, deleterious recessive alleles are accommodated without adverse effects through heterozygosity.

**Figure 2:**
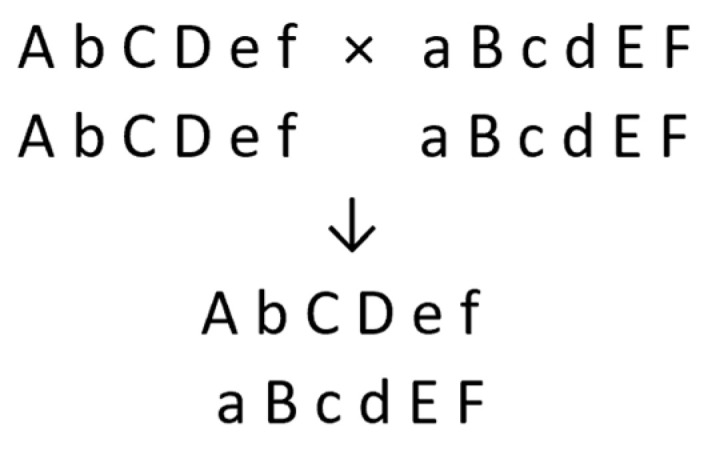
New diploid F_1_ hybrid cultivar from cross between two homozygous diploid parents (one pair of chromosomes shown, where small letters represent deleterious recessive alleles).

In contrast, when breeding diploid F_1_ hybrid cultivars for propagation through TPS, one needs to produce homozygous inbred lines so that the cross between a pair of lines is genetically uniform. For each of the 12 pairs of chromosomes, the female parent will have two copies of the same chromosome, and the male parent two copies of a different chromosome (Figure 2).

Despite being homozygous for some deleterious alleles, the inbred parents must have sufficient vigor and fertility for the maintenance and production of their hybrid.

The prerequisites for successful F_1_ hybrid breeding in plants are: (1) the ability to produce seed by self-pollination; (2) the ability to produce homozygous inbred lines with acceptable vigor and fertility, or sufficiently homozygous inbred lines to produce an F_1_ hybrid of acceptable phenotypic uniformity; (3) the ability to produce sufficient inbred lines for combinations to be found that are superior to existing cultivars, and to achieve this over cycles of inbreeding and crossbreeding for continued progress; and (4) the ability to produce large quantities of F_1_ seed for growing the hybrids commercially.

## 5. Successful Self-Pollination

Successful self-pollination of diploid potatoes was first made possible by the use of the dominant self-incompatibility inhibitor gene (*Sli*) found in *S. chacoense* by Hanneman [36], and mapped to chromosome 12, thus showing that it is independent of the *S* locus on chromosome 1 [37,38]. Jansky et al. [39] explained how inbred clone M6 (homozygous for *Sli*) was produced from self-compatible (SC) *S. chacoense* by seven generations of self-pollination, and found to be vigorous and fertile, as well as morphologically indistinguishable from *S. chacoense* plants that had not been inbred. It possesses a number of desirable agronomic traits, processing quality and resistance to *Pectobacterium carotovorum* soft rot and *Verticillium dahlia* wilt, as well as undesirable wild species traits, including unacceptably high levels of glycoalkaloids. Interestingly, it produced (small) tubers in a 14 h photoperiod, unlike many other wild species. Genomic information on M6 was provided when Leisner et al. [40] sequenced and assembled its genome. They anchored 508 Mb (million base pairs), out of the estimated 882 Mb from flow cytometry, into 12 chromosomes (pseudomolecules), and found that their genome annotation represented 37,740 genes. Analysis of single nucleotide polymorphisms (SNPs) across the whole M6 genome revealed 1,414,890 biallelic SNPs from a total of 208 Mb of assayable nucleotides, an SNP frequency of 0.68%, compared with 4.8% loci heterozygous, out of 8303 represented in the SolCAP SNP array [41]. There was enhanced residual heterozygosity on chromosomes 4 (1.73%), 8 (2.37%), and 9 (2.10%) compared with the overall figure of 0.68%. Endelman and Jansky [42] crossed M6 as the male parent with the male-sterile doubled monoploid *S. tuberosum* Phureja Group clone DM1-3 516 R44 (DM1-3), which was used to provide the first published sequence of the potato genome [43]. They self-pollinated the F_1_ to produce an F_2_ population for quantitative trait locus (QTL) analysis. Kaiser et al. [44] explored self-fertility and resistance to the Colorado potato beetle (*Leptinotarsa decemlineata*) in a diploid *S. chacoense* recombinant inbred line population derived from 308 F_2_ individuals after crossing M6 (self-compatible) and resistant accession USDA8380-1 (80-1, self-incompatible). Fifty-five F_5_ families were analyzed, and all individuals contained at least one copy of the *Sli* gene; however, this was not sufficient for selfed fruit and seed production. Loci on chromosomes 3, 5, 6, and 12 were identified as novel targets for self-fertility improvement, and a major QTL for foliar leptine glycoalkaloid biosynthesis and Colorado potato beetle resistance was mapped to chromosome 2. Self-compatibility and resistance to Colorado potato beetle were introgressed into diploid breeding material with desirable tuber traits. Thus, germplasm useful to practical breeders is emerging from such genetics research.

From 2008, Lindhout et al. [5,45] used the *Sli* gene for the production of diploid inbred lines that could be used to produce true F_1_ hybrid cultivars for maximum heterosis and genetic uniformity. They started by producing inter-species hybrids between diploid potato germplasm and a homozygous accession of the wild species *S. chacoense* carrying the *Sli* gene. The hybrids were extremely vigorous, and about half of them produced many berries upon self-pollination.

Clot et al. [46] found that the *Sli* allele (SC haplotype) is in fact widespread in the cultivated gene pool of the potato plant, including the tetraploid cultivars Garnet Chili, Irish Cobbler, Early Rose, Kennebec, Russet Norkotah, Sierra Gold, Yukon Gold, Snowden, Atlantic and Mountain Rose; with Garnet Chili, Irish Cobbler, and Early Rose probably, and Russet Norkotah definitely, possessing two copies of the *Sli* allele. They concluded that cultivar Rough Purple Chili, introduced into the USA in 1851 by Goodrich [47], is the origin of the SC haplotype. Interestingly, Haynes and Guedes [22] found that, in their long-day-adapted diploid population of Phureja/Stenotomum Groups, out of 42 clones evaluated, 32 flowered, and of these, 20 were successfully self-pollinated. Clot et al. [46] also developed KASP (Kompetitive allele-specific PCR) markers that can be used in breeding programs for the marker-assisted selection of self-compatibility (SC). Using a subset of six of these *Sli* KASP™ markers, Kaiser et al. [48] assessed the contribution of *Sli* to SC in the Michigan State University diploid germplasm, which represents diverse clones derived from multiple North American breeding programs. Although the *Sli* markers predicted SC in some germplasm, there were discrepancies, which emphasized the need to identify other genomic regions critical to SC and the role of the environment in the expression of genes involved in the SC reaction. For example, although M6 was confirmed as being homozygous for the SC *Sli* genotype at all six loci, the self-compatible clones 1S1 and DMRH-89, and a plant of *S. chacoense* accession PI 133664–40, were homozygous for the SI *Sli* genotype at the six loci.

Ma et al. [49] have confirmed that the self-compatible, heterozygous, diploid potato clone RH89-039-16 (RH) contains the *Sli* gene, and that it is capable of interacting with multiple allelic variants of the pistil-specific S-ribonucleases (S-RNases), and thus functions as a general S-RNase inhibitor to impart self-compatibility to RH and other self-incompatible potatoes. RH has a pedigree derived from dihaploidized tetraploid commercial cultivars.

Ye et al. [50] created self-compatible diploid potatoes through knock out mutations (loss-of-function) in the self-incompatibility *S-RNase* gene (at *S* locus) of the diploid self-incompatible *S.* *tuberosum* Phureja Group clone S15-65, using the CRISPR–Cas9 system. The growth vigor and plant morphology of the mutant lines did not differ from those of clone S15-65, indicating that they could be used directly for breeding. The percentage of plants in the selfed families without the Cas9 cassette varied from 3.6% to 24.5%, thus demonstrating that self-compatibility can be achieved without introducing any exogenous DNA. Using the same method, the researchers also obtained *S-RNase* mutants in two more *S.* *tuberosum* Phureja Group clones (S15-47 and S15-76) and two *S.* *tuberosum* Stenotomum Group clones (S15-48 and S15-107). Hence, improved diploid clones from a population improvement scheme could be made self-fertile for the purpose of inbreeding and crossbreeding to produce F_1_ hybrid cultivars. Enciso-Rodriguez et al. [51] also used the CRISPR–Cas9 system to generate targeted knockouts in conserved coding regions of the *S-RNase* gene. They achieved nine knockout lines (deletions/insertions) which transmitted self-compatibility to their progeny.

### Self-Pollination in Other Outbreeding Crops

Diploid hybrid breeding occurred much earlier in other outbreeding crops. The six examples given in my book on plant breeding [9] fell into three groups: (1) no incompatibility system to prevent self-pollination, (2) a sporophytic system, and (3) a gametophytic system.

Maize (*Zea mays*) naturally reproduces by wind cross-pollination, primarily, but not exclusively, as a result of having separate male and female flowers on the same plant (monoecious). However, it is also easy to self-pollinate, and hence proved suitable for both genetics and plant breeding research. The breeding research on the effects of inbreeding and crossbreeding in maize by East [52], Shull [53,54], and Jones [55] resulted in double-cross (DC) hybrids being grown in the USA from the 1930s, and single-cross (SC = F_1_) hybrids from the 1960s [56]. Onions (*Allium cepa*) are predominantly a cross-fertilizing, insect-pollinated, diploid species, although self-pollination occurs when the inflorescences of breeding material are simply bagged. F_1_ hybrids started to appear in the USA from the early 1950s, and in the Netherlands from the late 1960s [57], but are still to be developed in India [58]. Carrots (*Daucus carota*) are an outcrossing, insect-pollinated, diploid species with hermaphrodite flowers that are usually protandrous, although self-pollination is not restricted by incompatibility. Carrot hybrids started to become available in the 1970s and, since the 1980s, have been replacing open-pollinated cultivars worldwide [59]. 

Since the 1960s, F_1_ hybrid production has been the driving force behind the breeding of vegetable Brassicas (*Brassica oleracea*), such as Brussels sprouts, cabbage, calabrese/broccoli, and cauliflower. Self-pollination is normally prevented by a sporophytic self-incompatibility system, as was first demonstrated and explored by Thompson [60] in the context of fodder kale (*B. oleracea* var. *acephala*) breeding, which started at the former Plant Breeding Institute in Cambridge in 1951 [61]. Inbred lines can be produced and maintained by pollinating a bud with pollen from another flower on the same plant at least two days before the bud opens; a key difference from a gametophytic self-incompatibility system.

Rye (*Secale cereale*) is a diploid, cross-pollinated cereal with an effective gametophytic self-incompatibility system [62]. Dominant self-fertility genes were detected in various European germplasm [63,64,65] and transferred into breeding pools by repeated backcrossing. In Germany, hybrid breeding started around 1970 [66], and hybrid (single-cross × restorer synthetic composed of two inbred lines) cultivars predominated in most Western and Central European rye-growing areas by 2009 [67]. Sugar beet (*Beta vulgaris*) is another outbreeding crop in which self-fertilization is normally prevented by a gametophytic self-incompatibility system. Again, self-fertility was provided by a dominant gene (*S_f_*) [68]. In recent years, diploid sugar beet hybrids have become prevalent in Europe and elsewhere, most of which are three-way cross hybrids (2*x* CMS *mm* F_1_ × 2*x* N MM), where CMS is cytoplasmic-genetic male sterility, N is normal cytoplasm, and *mm* is the genotype required for the monogerm seed [69].

In conclusion, in the three crops with a gametophytic self-incompatibility system, potato, rye, and sugar beet, reliable and successful self-pollination required the discovery and use of a dominant self-fertility gene.

## 6. Self-Compatible Diploid Potatoes

In the USA, at Michigan State University, Alsahlany et al. [70] have shown that a population of self-compatible diploid potatoes can be developed through recurrent selection over a one-year cycle. In other words, diploid potatoes can be converted from a diploid outbreeding species, in which self-pollination is largely prevented by a gametophytic self-incompatibility system, into a diploid outbreeding species that can be self-pollinated, as in maize. This opens up the possibility of improving diploid potatoes by applying any of the population schemes used by maize breeders. The reader is referred to the paper by Alsahlany et al. [70] for details; some key points from this work are as follows. Four self-compatible (SC) clones, with a *S. chacoense* background, and eight self-incompatible (SI) ones, with introgressed disease and pest resistance traits of interest, were used to produce 50 offspring, from each of 24 SC × SI crosses, as the foundation population. Once underway, selected diploid germplasm was grown during winter in a greenhouse (16 h light/8 h dark photoperiod at 20–25 °C), checked for ploidy level (chloroplast counts in stomatal guard cells), and screened for self-compatibility. The pollen from self-compatible diploid clones was bulked and used to pollinate the selected female plants (SC and SI). Seed extraction and germination took place in spring, and seedlings were transplanted to the field in summer. Field selections based on maturity and tuber-related traits took place in autumn. The cycle was repeated annually over a five-year period. Self-fertility increased from 16% to 86% of the population, male sterility decreased from 32% to 0%, and non-flowering decreased from 19% to 3%. Single-nucleotide polymorphism (SNP) markers (4885) revealed a slight decrease in the frequency of heterozygous SNPs (34% to 30%) and a reduction in linkage disequilibrium; despite this, genetic diversity was maintained. There was an increase in earliness for vine maturity, as well as an increase in tuber yield from 430 g to 522 g over four cycles, which is a respectable 5% increase per year. The improved population can be used as the starting point for an inbreeding and crossbreeding program leading to F_1_ hybrid cultivars. However, it might first be desirable to select for increases in the frequencies of major genes for disease and pest resistance, as well as other traits, in order to increase the frequency of desirable homozygotes in the population. The question thus arises, how easy is it to produce homozygous inbred lines of potatoes?

## 7. Ability to Produce Homozygous Inbred Lines

One of the main reasons for conducting diploid F_1_ hybrid potato breeding for TPS is to achieve the genetic uniformity not possible in tetraploid potatoes with the methods available, as reviewed by Golmirzaie et al. [3], Ortiz [71], Simmonds [72], and Gopal and Ortiz [73]. Hence, it is important to know the expected level of homozygosity in diploids derived from a number of generations of self-pollination, and how this compares with what has been achieved, in practice, in potatoes and other crops.

### 7.1. Theory

It was Mendel [11] who realized that self-pollination (selfing) of a homozygote gives only homozygous offspring, whereas, on average, half the offspring of heterozygotes are also heterozygous, the other half being, on average, equal proportions of the two homozygotes, assuming equal viability and fertility in all plants. Mendel also devised a formula for the prediction of the expected proportion of heterozygous lines (*H_n_*) at a given locus after a given number of generations (*n*) of selfing, as follows:

*H_n_* = (½)*^n^H_0_*

where *H_0_* is the proportion at the start of the selfing program. For example, starting with 1 for the F_1_ generation, by F_6_ (five generations) we have 1/32, or 3.125%, and by F_9_ (eight generations) we have 1/256, or 0.39%. The prediction assumes equal viability and fertility of the three genotypes, i.e., equal contributions to the next generation. However, if the heterozygote makes a greater contribution than the two homozygotes, which make lesser but equal contributions (1−*s*, compared with 1 for the heterozygote), it can be shown that the proportion of homozygotes at equilibrium is 1/(2*s*). Hence, when *s* is greater than ½, complete homozygosity never results, and, when *s* = 1, the proportion of homozygotes produced is ½ [74,75]. An example of *s* = 1 is when there is a very tight repulsion linkage involving two recessive lethal alleles (*a* and *b*) so that homozygotes *Ab*/*Ab* and *aB*/*aB* prove lethal, and only the heterozygote *Ab*/*aB* survives to reproduce.

However, of more interest is the proportion of lines that are homozygous at all loci. Gale [76] shows in his book *Population Genetics* how this proportion can be calculated without knowing the number of loci through Fisher’s “theory of junctions” [15], as analyzed by Bennett [77]. The information required is the haploid chromosome number (*v*) of the plant species and its total map length (*L*), defined as half the number of chiasmata per nucleus, and measured in Morgans, together with an understanding of meiosis. It is assumed that, at the beginning, every locus is heterozygous. Then, after *n* generations, the proportion of lines homozygous at all loci is *e^-m^*, where *m* is the number of heterozygous tracts (stretches of chromosome that are not homozygous) averaged over lines, provided *n* is large enough for *m* to be small (i.e., ≥5 in Table 1).

*m* = *L*(½)*^n^* [2*n* + *v/L*]

For diploid potatoes, the respective values of *v* and *L* are 12 and 10 (approximately), and the results are shown in Table 1. In generation 0, the number of heterozygous tracts is 12, i.e., the haploid number of chromosomes. After eight generations of selfing, the average number of heterozygous tracts averaged over all lines is 0.671875, and 51% of lines are homozygous at all loci, whereas we saw above that, on average, 99.61% of lines are homozygous at any given single locus. After 12 generations, the values are 0.061523 and 94%, and after 16 generations, 0.005066 and 99%.

However, Gale [76] pointed out that the assumption that all genotypes at a given locus are equally viable and fertile cannot, in general, be justified, particularly for an outbreeding species. Indeed, he argued that a quantitatively accurate theory of the approach to homozygosity in outbreeding species is probably unattainable. In any population, deleterious mutations arise from time to time, although the majority will be recessive, for reasons discussed by Gale [76]. Hence, their harmful effects will not be apparent in heterozygotes, and their frequency will be determined by the balance of new mutations and selection against the mutant when homozygous (and genetic drift in small populations).

Textbooks on population genetics, such as that by Mather [75], give the derivation of the formula *q* = (*μ*/*s*)^½^ for a random mating population, where *q* is the equilibrium frequency of recessive allele *a*, *μ* is the frequency of mutations from *A* to *a*, and the relative fitness of *aa* is 1 − *s*, compared with 1 for *AA* and *Aa*. As *μ* is generally much smaller than *s*, it follows that *q* will be small and *q*^2^, the frequency of *aa*, will be very small.

In outbred populations, heterozygotes for the mutant will greatly outnumber homozygotes. However, although harmful recessive alleles at any given locus will be rare, individuals completely free of harmful recessive alleles at every locus on the chromosome (and hence in the whole genome) will also be very unusual in outbreeding species. Experimental results were summarized and discussed by Dobzhansky [78] in his 1970 book *Genetics of the Evolutionary Process*, in terms of lethal, semi-lethal, sub-vital, normal, and super-vital chromosomes of *Drosophila* species. When the corresponding homozygotes are formed through inbreeding, a marked reduction in fitness, known as inbreeding depression, occurs. Hence, inbreeding depression is a very widespread phenomenon in outbreeding species. In contrast, in inbreeding species, homozygous recessives appear much more frequently than in a random mating population, and, if harmful, will tend to be eliminated by natural selection so that little inbreeding depression is found.

It has also been argued that inbreeding depression is expected to be more severe in clonally propagated plants than in seed plants, and increases the difficulties in developing inbred lines. This is because a large number of deleterious mutations can accumulate over prolonged clonal propagation, although reproduction in potatoes in the Andes is probably a complex mixed sexual/clonal system; young plants in a field are a mixture of planted clones and recombinant genotypes from sexual reproduction [9]. Lian et al. [79] have studied genome-wide deleterious mutations among diploid landraces (20 Stenotomum Group clones), tetraploid landraces (8 Andigena Group clones), and modern tetraploid cultivars (20 commercial cultivars). The number of deleterious mutations increased going from the diploid landraces to the tetraploid landraces, with a further increase in the tetraploid cultivars. However, the tetraploid potatoes contained 41% fewer homozygous deleterious mutations and 140% more heterozygous ones than the diploid potatoes, and the proportion of heterozygous mutations was also higher in tetraploids. Within the cultivar group, 620 fixed deleterious mutations were detected. My interpretation of this is that dihaploids produced from tetraploid cultivars will have more deleterious mutations than diploid landraces. However, based on their research, Hoopes et al. [80] have expressed the view that access to haplotype-resolved tetraploid potato genomes with highly divergent haplotypes will aid in efforts to select dihaploids with the maximal combinations of advantageous alleles. Encouragingly, in their proof-of-principle paper for diploid F_1_ hybrid potatoes, Lindhout et al. [5] did not observe any lethal alleles among 24 informative SNP markers covering all 24 chromosome arms, although many loci were found with distorted segregations. However, when Zhang et al. [81] explored inbreeding depression in three F_2_ populations derived from three diploid clones (PG6226 = E and PG6235 = RH from Wageningen University, and PG6359 = C151 from CIP), they identified 15 genomic regions with severe segregation distortions due to gametic (4) and zygotic (11) selection. Five regions contained recessive lethal mutations, and four regions contained mutations affecting growth vigor. In addition to the alleles with substantial deleterious effects, there were numerous mutations that had minor effects on fitness.

### 7.2. Diploid Potatoes in Practice

As diploid potatoes are outbreeders, inbreeding depression and resistance to complete homozygosity at all loci is expected over the generations of self-pollination. Phumichai et al. [82] demonstrated that highly homozygous (>90%), seed-propagated, diploid potatoes could be obtained by crossing cultivated potatoes (e.g., Phureja Group) with an *Sli* gene donor (*S. chacoense*), followed by self-pollination for five generations (S_5_) (the detailed pedigrees are given in their paper). Serious loss of fertility was encountered, with only one of seven cross combinations reaching S_5_. Having started with 5040 plants from the seven families, just 43 out of 427 S_5_ plants produced selfed seeds. Furthermore, for RFLP (restriction fragment length polymorphism) and AFLP (amplified fragment length polymorphism) markers, the average percentage reduction in heterozygosity per generation (38.5%) was lower than the theoretically expected value (50%), indicating some resistance to homozygosity. None of the loci or chromosome sections were exclusively heterozygous in all of the advanced self-progeny.

More extensive results have come from the Solynta breeding and research program [45]. They started work in 2008 by producing inter-species hybrids between diploid potato germplasm from Wageningen University and a homozygous accession of the wild species *S. chacoense* carrying the *Sli* gene. The hybrids were extremely vigorous, and about half of them produced many berries upon self-pollination. However, the F_2_ plants showed weak growth, and many died in the field. Nevertheless, 10% of the surviving F_2_ plants proved self-compatible, and were used to produce the F_3_ generation. Undesired characteristics from the *S. chacoense* parent (abundant stolons; small leaves; and twisted, small, low-yielding tubers) were removed in a few generations. However, the first inbred lines, containing over 95% homozygous loci, produced few progenies, and the seedlings were extremely weak. Hence, new crosses were made between F_3_ and F_5_ inbred lines, followed by more intense selection over the subsequent selfed generations, and resulted in inbred lines with improved performance. By 2020, sufficient progress had been made for a single F_9_ line, named Solyntus, to be sequenced as the new standard reference in potato genome studies [83]. This sequence complements the updated version of the original genome sequence of DM1-3 516 R44, a doubled monoploid clone of the *S. tuberosum* Phureja Group [84]. Solyntus is a true diploid *S. tuberosum* that is highly homozygous, vigorous, and self-compatible. Its estimated genome size is 710 Mb, with 0.3% of the genome estimated to be heterozygous and 89.7% of the genome unique. However, when an SNP (single nucleotide polymorphism) rate of 200 SNPs per 30 kb was taken as the threshold for heterozygosity, 4379 out of 21,776 windows showed a signal above this threshold, which is equivalent to 20.1% of the genome still being heterozygous, and much higher than expected for an F_9_ inbred line. The authors attributed this to a combination of unnoticed and undesired outcrossing during the generation of Solyntus, and a preferential selection of heterozygotes in the selfing generations due to inbreeding depression.

Further insight into the task facing potato breeders comes from the haplotype-resolved genome analyses of the self-compatible heterozygous diploid potato RH89-039-16 (RH) by Zhou et al. [85]. RH has a pedigree from dihaploidized tetraploid commercial cultivars, such as Katahdin, Chippewa, and Primura. Comparison of its two haplotypes revealed ~2.1% intragenomic diversity, including 22,134 predicted deleterious mutations in 10,642 annotated genes. In 20,583 pairs of allelic genes, 16.6% and 30.8% exhibited differential expression and methylation between alleles, respectively. These were dispersed throughout both haplotypes, thus potentially complicating strategies to eradicate deleterious alleles or stack beneficial alleles via meiotic recombination. Analysis of a selfed RH population revealed that 25.7% of genomic regions (430.8 Mb) exhibited strong segregation distortion. Large-effect recessive deleterious mutations caused 71.4% of the SD regions. Six loci were identified in SD regions that affected either survival (white seedling 1 (*ws1*), abnormal rooting 1 (*ar1*), lethal allele 2 (*la2*)), or growth vigor (plant architecture 1 (*pa1*), plant architecture 2 (*pa2*), and weak vigor 1 (*wv1*)). Generally, large-effect deleterious mutations were relatively dispersed throughout the genome, and hence could relatively easily be removed by selection. However, two recessive detrimental alleles, *ws1* and *pa1*, were tightly linked in repulsion on the short arm of chromosome 1, and required larger populations and further analyses to find the desirable, but rare, recombinants.

**Figure 3:**
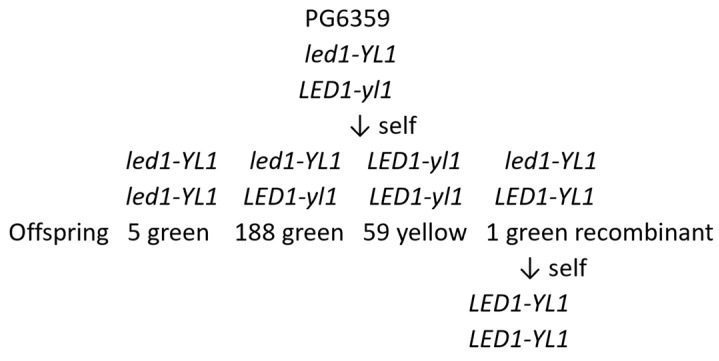
Recombinant among phenotypically green plants that broke the close linkage of two deleterious recessive mutations in repulsion phase at the end of chromosome 12, where *led1* is a large-effect deleterious mutation, and *yl1* is a deleterious mutation for chlorotic yellow leaves. Data from Zhang et al. [86].

Zhang et al. [86] have developed a method to produce pure inbred lines and vigorous F_1_ hybrids through genome design, which is far more precise and effective than phenotypic selection. The method involves four steps: (1) selection or creation, as starting materials, of self-compatible diploid clones with low genomic heterozygosity, (2) analysis of the S_1_ population of this starting material to identify the segregation distortion regions (SDs) and genetic loci carrying large-effect deleterious mutations or controlling agronomic traits, (3) production of highly homozygous inbred lines by continuous selfing and genome-assisted selection, to purge deleterious mutations and stack beneficial alleles, and (4) crossing the inbred lines, derived from different lineages, to produce F_1_ hybrids for evaluation. Using this method, they were able to select two (PG6359 and E86-69) out of four clones for a starting material that had lower heterozygosity and fewer deleterious mutations. They were able to detect segregation distortion (SD) in the selfed families (S_1_) of the four clones at 3, 2, 6, and 5 loci, respectively. The progeny of the latter two clones became very weak, and seldom flowered after two generations of selfing. Starting at S_1_, based on genetic analyses, they eliminated deleterious or undesirable alleles as well as stacking four beneficial alleles for self-compatibility, normal leaves, fertility, and yellow flesh. From the first heterozygous self-compatible clone PG6359, they were able to achieve average genome-wide homozygosity of 48.32% (range 19.30% to 74.76%) in S_1_, rising to 97.54% (range 91.79% to 99.94%) in S_5_. They were also able to use genomic analysis and selection to detect recombinants among phenotypically green plants that broke the close linkage of two deleterious recessive mutations in repulsion phase at the end of chromosome 12, where *led1* is a large-effect deleterious mutation and *yl1* is a deleterious mutation for chlorotic yellow leaves rather than normal green ones (Figure 3).

Inbred lines of similar homozygosity (in this case S_3_) were also produced from clone E86-69. Crosses were then made between the two sets of inbred lines. The F_1_ hybrids between homozygous inbred lines were very uniform, whereas those derived from heterozygous parental lines showed a lot of segregation for tuber traits. Furthermore, the inbred-line-based F_1_ hybrids showed strong heterosis in growth vigor and yield in the greenhouse, with the yield of the best F_1_ hybrid combination 3.38 times that of the mid-parent value. Mini-tubers from true seed plants were used to assess the hybrids in the field in Yunnan Province, China (24°43′ N 98°58′ E). Due to severe inbreeding depression, the inbred lines performed very weakly in the field, and only a few tubers could be harvested. This was despite the elimination of deleterious mutations of large effect from the inbred lines, thus indicating the presence of many homozygous deleterious mutations of minor or medium effects. In contrast, the F_1_ plants grew well, and the average yield was twice that obtained in the greenhouse, thus indicating that the inbred lines complemented each other for the deleterious genes. The estimated tuber yields of the hybrids was around 40 t ha^−^^1^, close to the yield of Lishu 6 (45 t ha^−1^), the major tetraploid cultivar grown in the region, with 65% coverage of the local cultivated area. Furthermore, due to the self-compatibility and recovered fertility, the F_1_ hybrids produced abundant fruits. The reader is referred to the paper for more details [86].

In future rounds of crossing and inbred line production, it may be possible to produce homozygous lines from F_1_ hybrids by anther/microspore culture, as reviewed by Veilleux [87]. Paz and Veilleux [88,89] managed to achieve this for diploid Phureja Group potatoes; however, the lines were all male-sterile, although of variable female fertility. The F_1_ hybrids mentioned above that produce abundant fruits may prove better starting material.

In summary, diploid F_1_ hybrid breeding has reached the point where it is a viable breeding method, as sufficient highly inbred lines of adequate vigor and fertility are now available for future breeding work and F_1_ hybrid production. Before looking ahead to future breeding work, it is worth briefly looking at experiences with other crops, particularly maize and vegetable Brassicas.

### 7.3. Maize in Practice

The comparison usually made for F_1_ hybrid potatoes is with maize, for which hybrid breeding is well documented in the literature, including books on plant breeding [9,90]. A few key points are as follows. Jones [91] reported the results of 30 generations of self-fertilization for three lines of maize. After five generations, height was reduced by 30%, with little change thereafter, whereas yield was reduced by 75% after 20 generations, with a further slight reduction of 4% by generation 30. Jones concluded that after 20 generations of self-fertilization the inbred lines were uniform and constant for all visible characters and homozygous for all loci that have any effect upon hybrid vigor. In practice, phenotypically uniform pure lines (inbred lines) could be developed from an open-pollinated cultivar by five to seven generations of self-pollination. Lindstrom [92] concluded from a survey of breeders in the USA that probably 100,000 lines had been tested by 1939 for at least three years, although very few were useful (about 2.4%). After 1940, the breeding emphasis switched to crossing the elite inbred lines now available in order to make further progress. Eventually, the inbred lines were strong enough for the commercial production of single-cross rather than double-cross hybrids from 1960, and, in 1990, they accounted for over 85% of production in the USA. By 2012, the world’s largest seed company, Pioneer Hi-Bred, was evaluating a global total of about 130,000 new experimental hybrids each year [93]. Moreover, in Europe, Limagrain tested 22,774 single-cross hybrids from 1266 parental inbred lines in multi-location trials over the period 1995 to 2002 [94]. Experiments in the USA Corn Belt, with historical single-cross hybrids and their inbred parents, provided evidence of a 122% increase (from 2.03 t ha^−1^ to 4.51 t ha^−1^) in the yields of the parental inbred lines available from 1930 to 1980, and a corresponding 74% increase (5.27 t ha^−1^ to 9.16 t ha^−1^) in the yields of their hybrids [56,95]. In other words, improvements in the inbred lines resulted in improvements in the hybrids, and the same should be true for F_1_ hybrid potatoes. Troyer [56] attributed these improvements to the continued selection for a tropical crop with a wide adaptative capacity to the temperate climate and cultural practices of the USA Corn Belt. Again, perhaps there are parallels with the journey of the potato from the Andes to becoming a global food crop.

### 7.4. Brassica Vegetables in Practice

The Brassica vegetables provide an example of the difference between outbreeding and inbreeding species. Swedes are a vegetable and fodder crop that belong to the species *B. napus*, along with oilseed and forage rape, and provide an interesting contrast to the *B. oleracea* vegetables, such as Brussels sprouts (*B. oleracea* var*. gemmifera*). *Brassica napus* (2*n* = 38, aacc) is the allotetraploid of *B. oleracea* (2*n* = 18, cc) and *B. rapa* (2*n* = 20, aa; formerly *B. campestris*) [96], two outbreeding species each with an effective sporophytic self-incompatibility system controlled at a single locus by a series of *S*-alleles [60,97,98,99]. In contrast, *B. napus* is normally self-compatible, and swedes (*B. napus* var. *napobrassica*) can be treated as an inbreeding species that is tolerant to inbreeding, presumably because the ‘a’ and ‘c’ genomes complement one another.

As early as 1966, Johnston [100], at the National Vegetable Research Station (NVRS) in the UK, demonstrated the commercial potential of F_1_ hybrid Brussels sprouts. The first F_1_ hybrid to be marketed from the program was cultivar Avoncross, with a marketable (good quality sprouts) yield 19% greater than its nearest competitor, cultivar Sherardian. The method developed for the field production of F_1_ hybrid seed was based on the self-incompatibility system; however, today, in the *B. oleracea* vegetables, cytoplasmic male sterility (CMS) is preferred, as briefly reviewed by Dixon [101] and Bradshaw [9]. In the 1980s, researchers at the University of Birmingham in the UK explored the possibility of producing inbred line cultivars of Brussels sprouts for ease of seed production, and to overcome the problem of contamination in F_1_ hybrids from sibs of the inbred parents [102]. Out of 2356 lines produced by single seed descent (SSD), none was superior to the F_1_ hybrid cultivar Gower for both marketable yield and sprout quality. The researchers concluded that in the short-to-medium term, inbred lines were uncompetitive compared with F_1_ hybrids.

In contrast, in swedes, Bradshaw et al. [103] had no difficulty in selecting two new cultivars, Gowrie and Lomond, from 1037 F_6_ families produced by SSD in a modest-sized breeding program in Scotland. Heterosis for yield does exist in swedes [104], and can be exploited in F_1_ hybrid cultivars produced from self-incompatible inbred lines [105], as proposed by Gowers [106,107,108]. However, pedigree inbreeding with selection from the best hybrid available produced cultivar Kenmore with an even higher dry matter yield [109]. Nevertheless, hybrids are preferred by commercial breeding companies as they are more profitable and have been available in the UK since 2010, although using cytoplasmic male sterility (CMS) rather than self-incompatibility (Gowers [108] from Kennedy of Elsoms, personal communication).

My conclusion from this section is that diploid potatoes, like Brussels sprouts (and maize), are likely to remain an outbreeding crop that can be self-pollinated rather than become an inbreeding crop, such as swedes. The reason for this is that it is difficult with an outbreeding crop to avoid producing inbred lines that are homozygous for (recessive) deleterious genes at certain loci, whereas it is much easier to produce hybrids that combine different chromosomes that complement one another at these loci.

Finally, it is worth pointing out the contrast between allotetraploid swedes (*B. napus*) and autotetraploid potatoes (*S. tuberosum*). The gametophytic self-incompatibility system breaks down in autotetraploids, at least when two different alleles are present in the pollen [110,111]. As a consequence, potatoes, like swedes, naturally reproduce by a mixture of self- and cross-pollination by insects [112,113]. However, potatoes suffer inbreeding depression upon self-pollination, and are thus regarded as an outbreeding species. Furthermore, being an autotetraploid, the approach to homozygosity via self-pollination is slow. Whereas seven generations of self-pollination are required for an initially heterozygous diploid organism to become 99% homozygous, assuming chromosomal segregation, it requires 27 generations in autotetraploids, which is not practical [15,114,115].

## 8. Cycles of Inbreeding and Crossbreeding to Produce Hybrids

We have considered the elimination of deleterious mutations of large effect during the selfing generations leading to inbred lines, and have seen the value of using molecular markers to aid this process. Now we assume that diploid F_1_ hybrid breeding in potatoes will progress through cycles of inbreeding and crossbreeding, as has happened in maize and other crops. It will, however, be important to consider the balance between incorporating new and desirable major genes and QTL (quantitative trait locus) alleles of large effect into new F_1_ hybrid cultivars, and accumulating desirable QTL alleles of small effect for improvements in quantitative traits where the number of loci is a key factor. We are now emphasizing positive selection for as many desirable (dominant) alleles of small effect as possible, rather than structural removal of many unfavourable (recessive) alleles of small effect [5]; nonetheless, the result is the same!

### 8.1. Introgression (Backcross) Breeding and Chromosome Segment Substitution Lines

**Figure 4:**
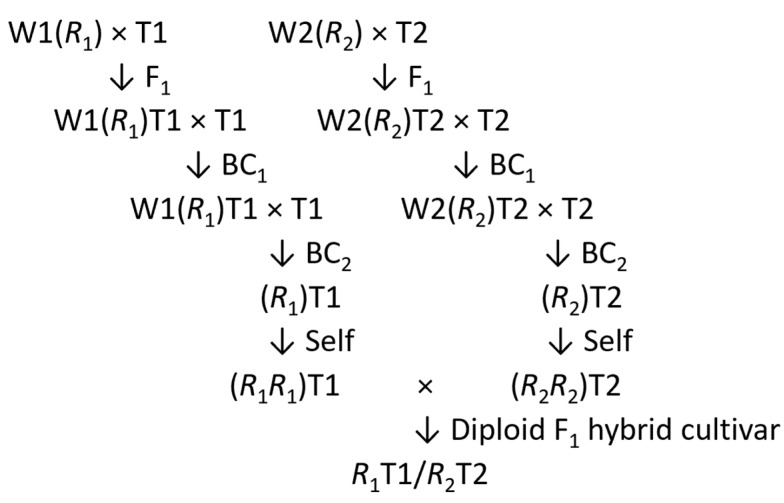
Introgression of two resistance genes *R_1_* and *R_2_* from two (heterozygous) wild species (W1 and W2) into two elite diploid Tuberosum (homozygous) inbred lines (T1 and T2), where selection in the F_1_, BC_1_, and BC_2_ generations is for the *R*-genes and, in the BC_1_ and BC_2_ generations, for as much of the Tuberosum genome as possible.

If desirable major genes and QTL alleles of large effect are not present in the elite germplasm being used, they can be introgressed (backcrossed) from potato wild relatives and landraces into elite lines as they emerge. Today, the introgressions can be performed quickly and efficiently through molecular-marker-assisted backcrossing, in which there is precise selection for the desired products of meiosis in each backcross generation [9,116,117]. When the desired allele is dominant, it only needs to be present in one of the parents of the hybrid, and hence two genes from the two parents can be brought together in the hybrid. With molecular-marker-assisted backcrossing, it is possible to achieve the desired objective in a modest-sized program in two backcross and one selfing generation. W1(*R_1_*) and W2(*R_2_*) are two (heterozygous) wild species with the two resistance genes, *R_1_* and *R_2_*, which may be homozygous or heterozygous. T1 and T2 are two elite diploid Tuberosum (homozygous) inbred lines. Selection in the F_1_, BC_1_, and BC_2_ generations is for the *R*-genes and, in the BC_1_ and BC_2_ generations, for as much of the Tuberosum genome as possible; ideally with no wild species genomes being left in the BC_2_ generation, apart from the *R*-genes. Finally, selfing the selected BC_2_ plants produces homozygotes for the *R*-genes (Figure 4).

Su et al. [118] have shown how this can be achieved in three years for two *R*-genes for resistance to *Phytophthora infestans*, the cause of late blight, namely Rpi-*tar*1 from *S. tarijense* [119] and Rpi-*vnt*1 from *S. venturii* [120,121]. The authors also explained that the method can be extended to stack more than two genes. Backcrossing can also be used to produce chromosome segment substitution lines, as explained in my book on plant breeding [9], with one of the examples taken from rice [122]. In an ideal scenario using potatoes, a cross between a wild species and an elite inbred line is followed by (probably) four backcross generations to the elite inbred line and two selfing generations. This is achieved using molecular markers in such a way as to dissect the wild species donor genome (W) into a complete set of non-overlapping homozygous chromosome segments in the genetic background of the elite Tuberosum (T) inbred line. For example, 120 segments (10 per chromosome) in 120 independently derived substitution lines (W_1_W_1_/T_2_T_2_/T_3_T_3_…, T_1_T_1_/W_2_W_2_/T_3_T_3_…, T_1_T_1_/T_2_T_2_/W_3_W_3_…T_118_T_118_/T_119_T_119_/W_120_W_120_). In practice, segments will be overlapping and of variable length; despite this, the effect of each genetically fixed segment on traits of interest, relative to the elite line, can be determined using appropriate trials to see if any of the substitution lines are superior to the elite line. Hence, using these backcrossing methods allows the fast, targeted, and predictable breeding of new potato cultivars.

Although alleles and chromosome segments of large effect can be stacked into an elite inbred line, if further improvements in commercially important traits require crossing to other elite lines, the genes of large effect will segregate, and combining more than, for example, eight of them into a new cultivar will require careful planning over a number of generations. Indeed, I would suggest that the introgression of genes, and combining them in a diploid F_1_ hybrid cultivar for TPS, is not that different to the introgression and combining of genes in a tetraploid cultivar for clonal propagation, once one thinks in terms of cycles of crossing and selection [35]. There will be difficulties and limitations in both processes, which is why genetic transformation and gene editing are attractive propositions in potato breeding [123]. It is now time to consider the quantitative traits and some simple theory.

### 8.2. Theory for Quantitative Traits

The theory and practice of diploid inbreeding and crossbreeding can be found in books on plant breeding and genetics [9,124], and is well-documented for maize [125,126]. Here, I first want to consider a very simple model for the means and variances of homozygous inbred lines and their single-cross (F_1_) hybrids as the frequencies of the desirable alleles increase as a result of selection. Starting with an additive-dominance model for a single locus, where *p* and *q* are frequencies of alleles *A* and *a*, we have the following:
Genotypes of F_1_ hybrids*AA**Aa**aa*Genotype frequency*p*^2^2*pq**q*^2^Frequency of *A* in F_1_ hybrids1½0F_1_ hybrid means*a**d*−*a*Genotypes of inbred lines*AA**AA**aa**aa*Genotype frequency*p*^2^*pq**pq**q*^2^Frequency of *A* in inbred lines1100Inbred line means*a**a*−*a*−*a*

Then, summing over loci (Ʃ), we have the following, where *m* is an arbitrary mean for all of the inbred lines when *p* = *q* = ½, *V_E_* is the plot-to-plot environmental variation in the assessment trials of hybrids and inbred lines, and *r* is the number of replicates in the trials:

Mean of F_1_ hybrid means

*M*_F1_ = *m* + Ʃ(*p* − *q*)*a* + Ʃ2*pqd*

Variance of F_1_ hybrid means

*V*_F1_ = Ʃ2*pq*[*a* + *d*(*q* − *p*)]^2^ + Ʃ(2*pqd*)^2^ + *V_E_/r*

Mean of inbred line means

*M*_I_ = *m* + Ʃ(*p* − *q*)*a*

Variance of inbred line means

*V*_I_ = Ʃ4*pqa*^2^ + *V_E_/r*

Responses to selection (*R*) can be determined from the following general formula [9].

*R =βS.dM/dp*

Here, *β* is the regression of allele ‘*A*’ frequencies on the phenotypic values of the assessed genotypes (covariance of allele ‘*A*’ frequencies and genotypic values divided by variance of phenotypic values of the assessed genotypes), *S* is the selection differential (mean of selected group minus mean of unselected population), and *dM/dp* is the rate of change in the population mean with the change in allele ‘*A*’ frequency.

Thus, the response to selecting the best inbred lines, seen in the next population of inbred lines produced by crossing the selected inbred lines in all combinations and then producing a new set of inbred lines from the crosses without any further selection, is as follows:

*R* = *βS.dM*_I_*/dp* = Ʃ2*pqa*/(Ʃ4*pqa*^2^ + *V_E_/r*) × *S* × Ʃ2*a* = Ʃ4*pqa*^2^/(Ʃ4*pqa*^2^ + *V_E_/r*) × *S*

In other words, the selection differential is multiplied by the heritability of the inbred line means, which can be determined from the assessment of the inbred lines. The predicted response to selection will give an indication of likely progress in practice. In contrast, the response in the population of F_1_ hybrids rather than in the next population of inbred lines cannot be determined so simply, as one needs to take into account the difference in population means.

*R* = *βS.dM*_F1_*/dp* = (Ʃ4*pqa*^2^ + Ʃ4*pqad*(*q* − *p*))/(Ʃ4*pqa*^2^ + *V_E_/r*) × *S*

We could also consider selecting among the F_1_ hybrids before starting the next cycle of inbreeding, but it is probably more useful to consider the following simple example of the effect of changes in favourable allele frequencies on the inbred lines and their F_1_ hybrids.

### 8.3. Simple Example of Inbreeding and Crossbreeding

**Figure 5:**
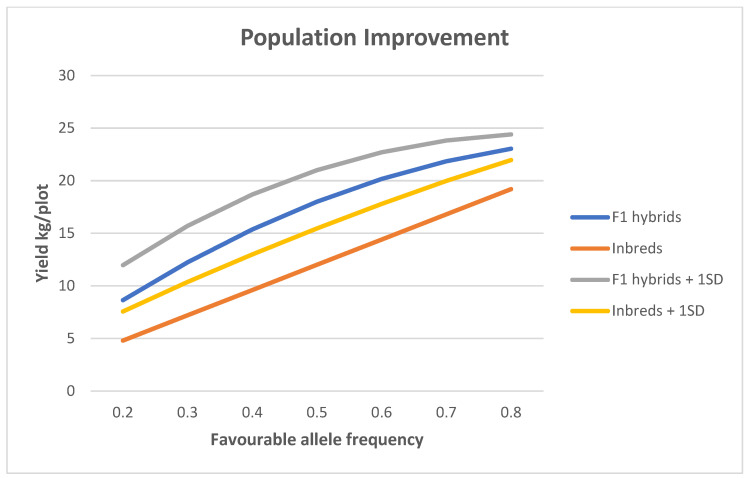
Improvements in mean of inbred lines and mean of F_1_ hybrids as frequencies of favourable alleles increase at 12 unlinked loci with complete dominance (*a* = *d* = 1), where mean (*m*) at *p* = ½ is set at 12 for inbred lines. Means plus one standard deviation (SD) are also shown.

If we assume complete dominance (*a* = *d* = 1) at each of 12 loci, one on each of the 12 pairs of chromosomes of diploid potatoes, we can produce the results shown in Figure 5 for a quantitative trait, such as yield, where the mean at *p* = ½ is set at 12 for the inbred lines.

As the frequency of the favourable allele at each locus increases (for simplicity, it is assumed that the increase is same at each locus), so does the mean and the mean plus one standard deviation for the inbred lines, and likewise for the hybrids. At a given allele frequency, one can consider the mean of the hybrids higher than the mean of the inbred lines as a result of heterosis, or the mean of the inbred lines lower than the mean of the hybrids as a result of inbreeding depression; the cause of both being the directional dominance for the higher yield. The degree of dominance (*d*/*a*, or dominance ratio) determines the magnitude of the effects (heterosis and inbreeding depression). The means plus one standard deviation indicates that the best hybrid will be better than the best inbred line.

**Figure 6:**
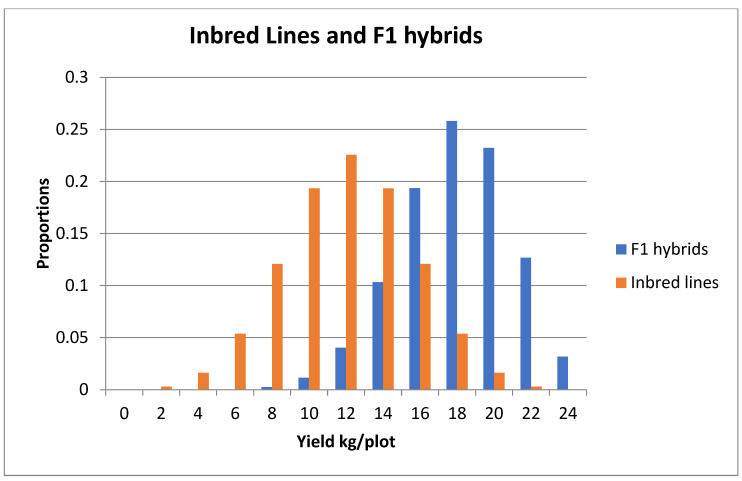
Inbred lines and F_1_ hybrids for 12 unlinked loci, each with complete dominance (*a* = *d* = 1) and *p* = ½ for favourable allele frequency, where mean (*m*) is set at 12 for inbred lines.

Figure 5 also shows that the differences between the four curves at a favourable allele frequency of 0.5 can be taken as fairly typical of the differences over the range of 0.2 to 0.8. Therefore, let us look in more detail at the variation among the inbred lines and among the F_1_ hybrids when the favourable allele frequency is *p* = ½ at all 12 loci, and there is no environmental variation (Figure 6). With 12 loci, there are 13 categories of inbred lines going from 0 (yield 0) to 12 (yield 24) loci homozygous for the advantageous allele. Likewise, there are 13 categories of F_1_ hybrids going from 0 (yield 0) to 12 (yield 24) loci either heterozygous or homozygous for the advantageous allele. The inbred lines show inbreeding depression with a mean of 12 compared with 18, the mean of the F_1_ hybrids. The variance (all additive) of the inbred lines (12) is greater than the variance of the F_1_ hybrids (9), which has an additive (6) and dominance (3) component. However, the higher variance does not compensate for the lower mean, so that 39.1% of the F_1_ hybrids have a yield greater than or equal to 20 compared with only 1.9% for the inbred lines. Furthermore, the frequency of the best inbred line is 1 in 4096 compared with (approximately) 130 in 4096 for the frequency of the equivalent best single crosses. However, the best inbred line is only 3.46 standard deviations from the mean of the inbred lines, and the best single crosses are only 2.00 standard deviations from the mean of the single crosses. In other words, with only 12 loci segregating, the best genotypes is quickly achieved and a selection limit is reached (e.g., yield plateau).

As the number of segregating loci (*n*) affecting the trait increases, the relative frequency of the best single crosses over the best inbred line increases (3^n^/2^n^, which for *n* = 12 is 531,441/4096). With 96 loci, at *p* = ½, the best inbred line is now 9.80 standard deviations from the mean of the inbred lines, and the best single crosses are 5.66 standard deviations from the mean of the single crosses. Hence, a number of cycles of inbreeding and crossbreeding will be required to achieve the best genotypes, as the selection differential in any modest-sized breeding program is likely to be around 2.00 standard deviations, and the heritability is likely to be less than the maximum of 1.00 [9]. These simple considerations demonstrate the importance of the number of segregating loci in determining the response to selection over a number of generations for a quantitative trait, and how quickly the selection limit is reached. It is also worth remembering that the ability to detect alleles of small effect is determined by the statistical power of the investigation, and has certainly been a limiting factor in potatoes as a result of the modest-sized populations used; for example, 49 to 272 genotypes in biparental mapping populations and 95 to 537 genotypes in panels for genome-wide association studies [35]. Hence, for polygenic traits, molecular markers are most likely to be used for genomic selection, where it is hoped that the correlation between true and estimated genomic breeding values will be as good as, or better than, the correlation between true breeding values and those simply estimated from the phenotypes, which is the square root of the narrow-sense heritability. 

### 8.4. Likely Practice for Diploid F_1_ Hybrid Potatoes

Breeding schemes for diploid F_1_ hybrid potatoes are still being developed, but some general features can be anticipated from both theory and other crops. Whatever the eventual worldwide scale of the breeding effort, it is clear that individual programs will potentially produce far too many inbred lines to assess all possible hybrid combinations. For example, for 512 lines there are 130,816 combinations and for 2048 lines 2,096,128 combinations. Hence, selection will be necessary during the inbreeding process, or among the inbred lines once they have been produced, before hybrids are produced from the selected lines and evaluated. Lines will need to be selected both for per se performance and for the general combining ability of quantitative traits in testcrosses, the latter starting in the earliest generation where it is practically possible [9]. The inbred line per se selection is necessary because the lines must have sufficient vigor and fertility to be grown as parents for the F_1_ hybrid seed production. Inbred line per se selection is most effective on additively inherited traits with high heritability, whereas testcross selection is more effective for traits such as yield, which display directional dominance and have low heritability [9]. Once elite inbred lines have been produced, further progress can be made by crossing them and repeating the whole process to produce a new generation of elite inbred lines, and so on in a cyclical fashion as discussed in the previous section on theory. The key to success will be deciding the balance between the selection for a number of major genes and QTL alleles of large effect and the selection for many QTLs of small effect (polygenic variation), and how these two types of selection will be integrated into an overall program aimed at producing cultivars for a target environment and target end use. The details will also depend on the method used for F_1_ hybrid seed production, and whether or not heterotic groups of germplasm are found.

## 9. Seed Production for Diploid F_1_ Hybrid Potatoes

We can conclude from earlier sections that sufficiently vigorous and fertile inbred lines of potato can now be produced for their maintenance and use as parents in diploid F_1_ hybrid production. Furthermore, despite a possibly greater mutational load, the task of producing such lines has proved of similar difficulty to that found in other outbreeding crops. We now need to consider how the F_1_ hybrid seed is going to be produced, as this has implications for how the inbred lines are sourced and selected. Earlier work on the commercial production of TPS in tetraploid potatoes is relevant, as reviewed by Gopal and Ortiz [73]. They emphasized the importance of the parental genotypes flowering for sufficient time and producing berries with enough seed, with genotype, daylength (photoperiod 14–18 h), and temperature (15–20 °C at night) as the main determinants. Furthermore, in the tropics and subtropics, high altitudes (above 1500 m) during summer are conducive to flowering and fruiting. These authors also provided information on factors affecting TPS quality, vigor, storability (dry seed), and dormancy (TPS is dormant at harvest).

Lindhout et al. [45] reported that the production of hybrid seed had mainly been achieved by hand pollination, where each successful hand pollination generated a berry with 50–150 (average 100) seeds and each plant produced 5–50 berries. Furthermore, the emasculation of the flowers was the time-consuming aspect of the hand pollinations. Van Dijk et al. [127] made some interesting calculations for a scenario where hybrid seed is sown in a greenhouse nursery and the resulting seedlings are transplanted to a field for tuber production. They envisaged 66,667 seedlings being transplanted per hectare of field to produce 525,000 seedling tubers. They calculated that 11.4 ha of net greenhouse nursery and hardening area is therefore needed to grow the Dutch ware potato and seed tuber export production, based on ware potatoes being grown from first-generation seedling tubers and the seed tuber export from third-generation seed tubers. In other words, 7600 pollinations (11.4 × 66,667 seeds/seedlings ÷ 100 = 7600 pollinations/berries) would need to be performed, possibly from as few as 200 plants if each produced 38 flowers on average. Other values will probably apply to other situations, but the calculations give an indication of what might be involved.

Another crop where F_1_ hybrids are largely produced by hand pollinations is the tomato (*Solanum lycopersicum*). It is an inbreeding glasshouse and field crop in which the older, open-pollinated landraces were largely replaced by hybrids during the second half of the 20th century [128]. The hand pollinations are frequently performed in areas with abundant cheap labour, such as east Asia, and can be justified by the high price of hybrid seed [129]. Emasculations, but not hand-pollinations, could be eliminated by the use of genic male sterility [130]. It is also worth noting that the two largest producers of tomatoes are China and India, the same as for potatoes, and the two countries where Chilver et al. [4] thought that a small but sustained TPS breeding effort could be justified. In contrast, F_1_ hybrid breeding in all of the outbreeding crops mentioned earlier from Bradshaw [9], required a method of cheap, large-scale hybrid seed production, with cytoplasmic male sterility eventually being the preferred option.

Maize has separate female and male flowers so that hybrid seed can be produced in isolated crossing blocks (seed fields) in which the female parent is detasseled, and in which the most common planting pattern is one row of pollen parents to four rows of seed parents [131]. Since 1971, mechanical detasselers have been widely used, although hand detasseling is still required to remove those tassels remaining on missed plants [93]. Today, the other common method of hybrid seed production is the use of newer forms of genetic-cytoplasmic male sterility (CMS), after the original Texas (T-) cytoplasm proved susceptible to the toxin produced by race T of *Bipolaris maydis* (formerly *Helminthosporium maydis*), the cause of southern corn leaf blight [132,133]. CMS involves a three-line system where one line (A) is the male-sterile, female parent, the second line (B) is used to maintain the sterility, and the third line (R) is the male parent that restores the fertility of the hybrid through a dominant restorer gene(s). Details can be found in textbooks on plant breeding, such as my own [9]; the important point, however, is that once a source of CMS has been found, all male-fertile germplasm is either a maintainer or a restorer with respect to the source. Hence, in breeding F_1_ hybrids using CMS, one is seeking maximum heterosis (hybrid vigor) between maintainer and restorer inbred lines based on their combining abilities. Hybrid rye is also based on cytoplasmic male sterility, and a restorer as pollinator [67].

The other diploid outbreeding crops considered by Bradshaw [9], as examples of F_1_ hybrid breeding, were the *B. oleracea* vegetables (e.g., Brussels sprouts), onions, carrots, and sugar beet. Today, hybrid seed production for all of these crops uses CMS, but there is no need for a restorer as the hybrids are non-flowering vegetable or beet crops; in fact, premature bolting is highly undesirable. Nevertheless, the use of CMS in testcrosses, followed by assessment of male-sterility, divides the germplasm into maintainers and restorers. Onions provide a good early example where a source of CMS (CMS-S) still used today was first discovered in 1925, and whose genetics was worked out by Jones and Clarke [134]. CMS-S results from a combination of male-sterile cytoplasm (S) with a homozygous recessive genotype *msms* at a nuclear male-fertility-restoration locus (*Ms*), so that male-sterile plants have the composition S-*msms*. Male-sterile plants can be seed-propagated by crossing S-*msms* (female) with N-*msms* (male) and harvesting seed from the female parent. They can then be used as the female parent in hybrid production. The nuclear genotype of the maintainer line (male) can be very similar to that of the female parent as a result of repeated backcrossing during maintenance. A high frequency of the *Ms* allele in germplasm of particular interest to a breeder makes finding a maintainer more difficult. As an onion crop does not flower, the unrelated male inbred parent used in hybrid production can have genotype N-*MSMS* or N-*msms* (i.e., it does not have to be a restorer), or even S-*MSMS.* Details of a hybrid onion breeding scheme can be found in the review by Shigyo and Kik [57].

Male sterility could be used in diploid F_1_ hybrid potato production in the same way as just described for onions, and this prospect will probably stimulate more research into the topic. The types of cytoplasm found in potatoes and their wild relatives, and their associations with male sterility and nuclear restorer genes, have been briefly reviewed by Ortiz and Mihovilovich [27] and by Bradshaw [35], and more extensively by Anisimova and Gavrilenko [135]. Seven distinct types of cytoplasm (A, M, P, T/β, W/β, W/γ, and W/α (= D)) have been found based on combinations of chloroplast and mitochondrial (α, β, γ) DNA. The T/β, W/γ, and D cytoplasms in Tuberosum Group potatoes, *S. stoloniferum* and *S. demissum*, respectively, are commonly associated with male sterility, unlike the A and P cytoplasms in Andigena and Phureja Group potatoes, respectively. Attempts may also be made to exploit the segregation at the *Sli* locus for self-compatibility/incompatibility in diploid F_1_ hybrid potato production. In the meantime, the important point is that the use of hand pollination means that there are no constraints on the use of diploid inbred lines of potatoes as female or male parents. It is too early to say if heterotic groups will emerge in the diploid potato germplasm. A heterotic group (e.g., 1) is the germplasm that, when crossed with the germplasm from another heterotic group (e.g., 2), exhibits a higher degree of heterosis than when crossed within its own group; thus, 1 × 2 hybrids are superior to both 1 × 1 and 2 × 2 hybrids. Heterotic groups in maize were established empirically, and their origin is not as clear-cut as once thought, as can be seen in the results of van Heerwaarden et al. [136]. Where heterotic groups exist, selection during inbreeding within each heterotic group is for combining ability with the other heterotic group, using a sample of bulked pollen or the best inbred lines from that group. The establishment of heterotic groups in maize resulted in the development of reciprocal recurrent selection schemes designed to improve the cross between two populations from different groups. A summary of results and theory can be found in the review by Hallauer and Carena [125].

## 10. Progress and Success in Diploid F_1_ Hybrid Potato Breeding

Assessing the progress and success in diploid F_1_ hybrid potato breeding is not quite as obvious as one might first think. All of the crops considered in this paper, apart from potatoes, are (true) seed-propagated, where F_1_ hybrid cultivars replaced open-pollinated ones. The hybrids were genetically more uniform and higher yielding; it was easier to combine a number of desirable traits in a hybrid; and there were commercial incentives, as hybrids do not breed true, and hence their seed cannot be saved for propagation. Thus, in maize, as we have seen, open-pollinated cultivars were replaced by double-cross hybrids, which in turn were replaced by single-cross hybrids, and there were also improvements in maize agronomy [56,137]. The yields of open-pollinated cultivars had been virtually static in the USA from 1865 to 1927, with the highest average around 1.88 t ha^−1^ [56,137]. Then, dramatic and steady increases occurred, which saw yields reach 8.78 t ha^−1^ by 1995, with about half the increases resulting from improved agronomy and half from the change to hybrids and improvements in those hybrids. In Germany, to take another example, by 2009, the best rye hybrids were outyielding the best open-pollinated cultivars by 15–20% [67]. Accounts and achievements in hybrid breeding of other crops are best sought in the most current reviews on breeding these crops, such as those I used in writing my book on plant breeding, albeit published six years ago [9].

The obvious comparisons for diploid F_1_ hybrid potatoes would be the current cultivars being grown for propagation through TPS. However, when Chilver et al. [4] reviewed on-farm profitability and prospects for TPS, there were no diploid F_1_ hybrids. Furthermore, they were considering the net benefits of replacing vegetative propagation of potatoes with TPS technologies. Now, the first assessments of diploid hybrids are also being made in comparison with tetraploid cultivars for tuber propagation. Three examples will suffice.

The first scientific paper from the Solynta breeding and research program on the contribution and stability of yield components of diploid hybrid potatoes came from crosses made in the winter of 2015/2016 between inbred parents that had been selfed for five to seven generations [138]. Seedling tubers were produced in 2016. Then, in 2017, 65 out of 572 hybrids that had been selected for uniformity, but not yield, were compared with a range of 19 tetraploid cultivars in trials at five sites, three in the Netherlands, one in Belgium, and one in France. The diploid hybrids yielded, on average, between 16 and 52 t ha^−^^1^, with an overall mean of 31.4 t ha^−1^, and the tetraploid cultivars between 52 and 101 t ha^−1^, with an overall mean of 72.1 t ha^−1^. Hence, on average, the diploid fresh weight yield was 43.6% that of the tetraploids, mainly as a result of the lower weight of individual tubers (71 vs. 177 g on average). The diploids had a higher number of stems (25.6 vs. 14.9 stems m^−2^) but fewer tubers per stem (1.8 vs. 2.9 tubers), resulting in approximately equal numbers of tubers per unit area (45.2 vs. 43.5 tubers m^−2^). 

Another early result from the Solynta team is also worth mentioning. De Vries et al. [139] reported on the potential of F_1_ hybrid potato for the tropical highlands of East Africa, where clean planting material is a problem, and hence TPS is an attractive proposition. In 2016, they trialed 10 representatives (out of 203) of their first experimental hybrids (crosses between F_4_ lines made in winter 2014/15) in East Africa at 1680 masl. Seedlings were raised from botanical seeds, and six weeks later transplanted to the field into irrigated ridges. Tubers were harvested four months later when all of the plants had died. The diploid hybrids yielded between 8 and 29 t ha^−1^ compared with average yields of potatoes in East Africa of 5 to 14 t ha^−1^. The best hybrid had yielded 26 t ha^−1^ in a Netherlands trial the previous year. These are just preliminary results, but indicate the potential of diploid hybrids in East Africa and the yield to be made up on tetraploid cultivars in the Netherlands that yield 35 to 40 t ha^−1^. Lindhout et al. [45] have put forward views on the social and economic requirements for making diploid hybrids a reality for East Africa.

As mentioned earlier, in Yunnan Province in China, Zhang et al. [86] estimated tuber yields of their best hybrid at around 40 t ha^−1^, close to the yield of Lishu 6 (45 t ha^−1^), the major tetraploid cultivar grown in the region, with 65% coverage of the local cultivated area. Furthermore, due to the self-compatibility and recovered fertility, the F_1_ hybrids produced abundant fruits.

Although the first diploid F_1_ hybrids to be assessed were lower-yielding than clonally propagated tetraploid cultivars, Jansky and Spooner [7] have argued that diploids may not be intrinsically lower-yielding than tetraploids. Hence, it may be possible to breed equally high-yielding diploid F_1_ hybrids, but more experimental results are required before reaching a conclusion. One indication will come from population improvement programs conducted over short cycle times, such as that in the USA at Michigan State University, where Alsahlany et al. [70] achieved a yield increase of 21% over four annual cycles of selection in a population of self-compatible diploid potatoes; nevertheless, it is not clear how their yield compares with tetraploid potatoes. It is, however, worth pointing out that the breeding of diploid F_1_ hybrids for TPS propagation, and the breeding of tetraploid cultivars for clonal propagation, both rely on additive genetic variation for continued progress in improving quantitative traits over cycles of crossing and selecting. In any given cycle, however, the best genotype selected, whether a hybrid or clone, exploits both the additive and non-additive genetic variation available in the cycle. Finally, both breeding methods can incorporate marker-assisted selection of major genes and QTL alleles of large effect, as well as genomic selection for polygenic traits, and both can incorporate gene editing and genetic transformation.

Further research is also required to determine whether or not TPS crops are intrinsically lower-yielding than crops grown from more nutrient-rich tubers. I would, however, suggest that the major issue facing potato breeders concerns the target environments for, and end users of, the cultivars that will emerge from their breeding programs. Which makes more practical sense in a given situation, a diploid F_1_ hybrid cultivar for TPS propagation, or a tetraploid cultivar for propagation from tubers? Hence, I will finish with some practical considerations.

### 10.1. Registration, Multiplication, and Protection of New Cultivars

All potato breeding programs start with hybridizations that will either be cross-pollinations or self-pollinations. The difference between diploid F_1_ hybrid cultivars for TPS propagation and cultivars for clonal propagation lies in their maintenance and production. For both types of cultivars, there must be a definitive stock, against which further multiplications can be checked. Here, I want to concentrate on the biology; however, a breeder cannot ignore the legal requirements in any territory (country or group of countries) for the registration, multiplication, and protection of new cultivars, nor the political, social, and economic context of the work. Details can be found in my books on plant and potato breeding [9,35]. Beumer et al. [140] have explored such matters in the context of the governance of genetic resources in potato breeding, with experience in the Netherlands as an example. They explain the concept of “the commons” as a shared resource, co-governed by a community of users according to their rules and norms. Interestingly, Beumer and Stemerding [141] have argued that a new kind of partnership between private sector breeding companies, public sector institutes, and farmers may be needed for smallholder farmers in poorer countries to benefit from diploid F_1_ hybrid potato breeding, and thus help to ensure food security.

Diploid F_1_ hybrid cultivars are produced by crossing two inbred lines that can be maintained as true seeds by self-pollinations, and which commercial companies will want to keep private and secure to protect their investment [140]. The cultivar is available as true seeds for sowing, and results in plants that produce tubers. Cultivars for clonal propagation are produced and maintained as tubers, but ideally should start from pathogen-free micro-plants that were derived from tuber sprouts, maintained under sterile laboratory conditions. In vitro pathogen-free nuclear stocks of cultivars can be maintained. The cultivar is available as fully rooted in vitro plantlets.

### 10.2. Planting Material for Farmers

A diploid F_1_ hybrid cultivar, therefore, starts life as true potato seed from which plants are grown, whereas a clonally propagated cultivar starts life as fully rooted in vitro plantlets. The success of both types of cultivars will partly depend on how they are made available to farmers for planting, or, in other words, the cost and quality of the seed system used to provide the planting material.

Low-income countries are characterized by informal seed tuber systems with very low use of certified seed, often below 10% [142], where farmers’ rights to save and trade their own seed tubers can result in planting material with a high level of tuber-borne diseases. The result is usually low-yielding, poor-quality crops. In Southwestern Uganda, Priegnitz et al. [143,144] found some benefits from positive seed tuber selection for three generations over the farmers’ traditional method of visual selection for tubers from the bulk of the potato harvest. Under these circumstances, TPS could have a lot to offer in terms of clean planting material.

In contrast, high-income countries are much more likely to have a commercial seed tuber industry, cultivar protection, and harmonization of standards. A typical seed tuber production scheme is likely to start with fully rooted in vitro plantlets (transplants) from which mini-tubers are commercially produced in a greenhouse or screenhouse with high health status [35]. The transplants can be grown in soil, but higher numbers of uniform tubers (up to 40 compared with 2 to 5) can be achieved from frequent harvests in aeroponic, hydroponic, or nutrient film cultures. Mini-tubers are then multiplied in the field for the number of generations required to meet demand. Virus-free seed tuber potato production started in China in the 1970s, and is now increasing as larger enterprises become involved [145]. The development of seed production in India since 1949 has been described by Singh et al. [146], including ‘the seed plot technique’ (separate plots for seed) from 1970, and more recently a hi-tech system where micro-plants are used to produce mini-tubers, either via micro-tubers, or from in-vitro plantlets, particularly in aeroponic systems, all under protected net house conditions [147]. Kreuze et al. [148] have described an effective low-cost system in Brazil for producing mini-tubers from potato sprouts from virus-free seed potatoes. Finally, Parker and Reddy [149] have described a system of rapid multiplication in Kenya that uses apical cuttings from tissue culture micro-plants to produce plants in a screenhouse for planting in the field to produce tubers. A second field generation of multiplication can provide enough seed tubers for planting crops.

TPS cultivars and production systems will need to prove superior to those achieved with conventional cultivars through modern methods of rapid multiplication of ‘disease-free’ seed tubers, if they are to replace them. Before the advent of diploid F_1_ hybrid cultivars, breeders and seed producers already had experience producing planting material for farmers from TPS. The methods fell into two categories; those in which there was no pre-propagation before commercial production, and those in which a multiplication phase intervened [72]. The first included the direct drilling of the seed, and also transplanting seedlings, as conducted with many vegetable crops. Transplanting proved the superior method, and became the preferred method by about 1980, although pelleted and primed seeds are possibilities for the future. Recently van Dijk et al. [127] showed that hilling of seedlings grown from hybrid TPS, and transplanted from a greenhouse nursery, does not enhance tuber yield under normal Dutch agronomic conditions, but can change tuber size distribution towards smaller tubers. However, back in the 1980s, a multiplication phase looked even more attractive. Potato plants grown in dense nurseries, at about 100 plants per square metre, produced small but usable mini-tubers, with an average size of 10–15 g, that could be chitted before being planted. Only 750 kg of seed tubers with an average size of 15 g are required to plant 1 ha of potatoes [73]. Hence, we are in a situation where all types of potato cultivars could be grown from mini-tubers. However, the production of mini-tubers from TPS may prove cheaper than from micro-plants, and will have the flexibility of starting production where and when required, and in appropriate numbers [45]. Furthermore, fewer rounds of multiplication may be required to satisfy the commercial demand for planting material, ideally just one round, and then release to commercial farmers. Lindhout et al. [45] also considered the possibility of a new cropping system that would allow commercial ware production from transplants, citing results from Kenya as an early success. van Dijk et al. [127] also considered schemes that allow ware tubers to be harvested in one to three seasons after sowing hybrid TPS.

## 11. Conclusions

Diploid F_1_ hybrid potatoes provide the means to grow genetically uniform cultivars from true potato seed (TPS). Diploid germplasm is available in the form of potato wild relatives, landraces, cultivars, and dihaploids from tetraploid cultivars. Reliable self-pollination of diploid potatoes is possible through the use of the dominant self-incompatibility inhibitor gene *Sli*, initially found in *S. chacoense*, but later also in the cultivated gene pool. Self-compatibility can also be achieved through knock-out mutations in the *S*-locus that normally controls gametophytic self-incompatibility. Diploid potatoes can, therefore, be converted from an outbreeding species, which cannot be self-pollinated, into one which can, but are unlikely to become an inbreeding crop.

Inbred lines of potatoes can be produced by a number of generations of self-pollination, with sufficient levels of homozygosity, vigor, and fertility, for F_1_ hybrid breeding and production, despite inbreeding depression and resistance to complete homozygosity. Molecular markers can be used to detect and remove recessive deleterious mutations of large effect, including those in tight repulsion linkage, and also to stack beneficial alleles. Genes (alleles) and chromosome segments of large desirable effect can be introgressed from wild relatives and stacked into elite inbred lines by backcrossing, but will segregate when the elite lines are used in further cycles of inbreeding and crossbreeding. The number of cycles of inbreeding and crossbreeding required to produce the best possible inbred lines and F_1_ hybrids for quantitative traits increases with the number of segregating loci affecting the trait. Breeding schemes used for diploid F_1_ hybrid potatoes will depend on the method used for F_1_ hybrid seed production, and whether or not heterotic groups of germplasm are found, but all will require the inbred lines to be selected for vigor, fertility, and combining ability for quantitative traits. Currently, diploid F_1_ hybrids are lower-yielding than tetraploid clones as a result of the lower weight of individual tubers; however, the likely relative rates for future improvements are not clear, although both will depend on the additive genetic variation in the germplasm.

Seed production is currently achieved by hand pollinations, and hence poses no constraint on the use of inbred lines as female or male parents, but cytoplasmic male sterility could become the preferred option for cheap, large-scale seed production. In deciding between diploid F_1_ hybrids for TPS and tetraploid clones in any given situation, the economics and practicalities of producing clean planting material is a major consideration. Diploid F_1_ hybrids start from true seeds, whereas clones start from pathogen-free micro-plants. Both types of cultivars require a “seed system”, whether true seed or seed tubers, to deliver planting material to farmers. This can be true seeds, young plants, or mini-tubers for F_1_ hybrids, and young plants or mini-tubers for clones, with further limited field multiplications to meet demand, which should be fewer from TPS. As potato is a major food crop, breeding will continue to operate in a social, economic, political, and legal context in which food security is both a practical and moral issue. 

## Figures and Tables

**Table 1:** Approach to homozygosity over 16 generations of selfing in diploid potatoes using the method of Gale [76].

Generation (*n*)	Mean Number of Heterozygous Tracts (*m*)	Proportion of Lines Homozygous at All Loci
0	12.000000	0
1	16.000000	very small
2	13.000000	very small
3	9.000000	small
4	5.750000	small
5	3.500000	0.0302
6	2.062500	0.1271
7	1.187500	0.3050
8	0.671875	0.5108
9	0.375000	0.6873
10	0.207031	0.8130
11	0.113281	0.8929
12	0.061523	0.9403
13	0.033203	0.9673
14	0.017822	0.9823
15	0.009521	0.9905
16	0.005066	0.9949

## Data Availability

No new data were created or analyzed in this study. Data sharing is not applicable to this article.
